# Structural variation provides novel insights into dog domestication

**DOI:** 10.1093/nsr/nwy099

**Published:** 2018-11-06

**Authors:** Fuwen Wei

**Affiliations:** Fuwen Wei CAS Key Lab of Animal Ecology and Conservation Biology, Institute of Zoology, Chinese Academy of Sciences, China Recommender of NSR

The article ‘Structural variation during dog domestication: insights from grey wolf and dhole genomes' by Wang *et al*. presented the first and comprehensive study of the evolutionary dynamics of structural variation of dogs based on three assembled Canine genomes [1].

Structural variations (SVs) play a prominent role during the domestication of animals in phenotypic evolution, disease susceptibility and environmental adaptation. As the first animal to be domesticated, the dog is of great interest and significance to human society [2–4]. Nevertheless, SVs during dog domestication are still poorly understood because of the lack of a genome assembly of the dog's wild ancestors.

Wang *et al*. report a high-quality *de novo* grey wolf (*Canis lupus*) genome and a dhole (*Cuon alpinus*) genome, respectively, and build a map of dog SVs through whole-genome alignments. Comparative analysis reveals that the dog genome has accumulated the LINEs and SINEs, providing basic genetic materials for domestication. In-depth analysis of the genomes explores the evolutionary tracks for energy metabolism, neurological processes and the immune system. In particular, one dog-specific insertion fully covered a novel copy of gene *AKR1B1* that is involved in *de novo* fatty acids synthesis and carbonyls detoxification. Transcriptomic analysis showed that the new copy is expressed in the small intestine at a high level.

This study indeed demonstrates that SVs are of great importance and worth paying more attention to during domestication in the genomic era. It also revealed that a change in dietary composition in dog domestication has sculptured not only on the genetic basis of starch-rich diets, but also lipid biosynthesis and carbonyls detoxification of the agricultural revolution. It is therefore a significant step forward in the genetic hunt for the evolution of complex traits.

## References

[1] Wang G-D , Shao X-J, Bai B Natl Sci Rev 2019; 6: 110–22. 10.1093/nsr/nwy076 PMC8291444 34694297

[2] Wang G , Zhai W, Yang H Cell Res 2016; 26: 21–33. 26667385 10.1038/cr.2015.147 PMC4816135

[3] Ostrander EA , Wayne RK, Freedman AH Nat Rev Genet 2017; 18: 705–20. 28944780 10.1038/nrg.2017.67

[4] Freedman AH , Wayne RK. Annu Rev Anim Biosci 2017; 5: 281–307. 27912242 10.1146/annurev-animal-022114-110937

